# Atorvastatin added to interferon beta for relapsing multiple sclerosis: a randomized controlled trial

**DOI:** 10.1007/s00415-012-6513-7

**Published:** 2012-05-09

**Authors:** Christian Philipp Kamm, Marwan El-Koussy, Sebastian Humpert, Oliver Findling, Ferdinand von Bredow, Yuliya Burren, Guido Schwegler, Dagmar Schött, Filippo Donati, Martin Müller, Norbert Goebels, Felix Müller, Johannes Slotboom, Barbara Tettenborn, Ludwig Kappos, Yvonne Naegelin, Heinrich Paul Mattle

**Affiliations:** 1 University Department of Neurology, Inselspital, Bern University Hospital and University of Bern, Freiburgstrasse, 3010 Bern, Switzerland; 2 Institute of Diagnostic and Interventional Neuroradiology, Inselspital, Bern University Hospital and University of Bern, Bern, Switzerland; 3 Department of Neurology, Cantonal Hospital Aarau, Aarau, Switzerland; 4 Department of Neurology, Cantonal Hospital St. Gallen, St. Gallen, Switzerland; 5 Department of Neurology, Spitalzentrum Biel, Biel, Switzerland; 6 Department of Neurology, Cantonal Hospital Lucerne, Lucerne, Switzerland; 7 Department of Neurology, University Hospital Zurich, Zurich, Switzerland; 8 Department of Neurology, Cantonal Hospital Thurgau, Muensterlingen, Switzerland; 9 Department of Neurology, University Hospital, University of Basel, Basel, Switzerland

**Keywords:** Multiple sclerosis, Atorvastatin, Interferon beta, Randomized clinical trial

## Abstract

Statins have anti-inflammatory and immunomodulatory properties in addition to lipid-lowering effects. The present study evaluated the effect of atorvastatin added to interferon beta-1b in multiple sclerosis (MS) in a multicenter, randomized, parallel-group, rater-blinded study performed in eight Swiss hospitals. Seventy-seven patients with relapsing-remitting MS started interferon beta-1b every other day. After 3 months, they were randomized 1:1 to receive atorvastatin 40 mg/day or not in addition to interferon beta-1b until month 15. The primary endpoint was the proportion of patients with new lesions on T2-weighted images at month 15 compared to baseline at month three. At study end, the proportion of patients with new lesions on T2-weighted images was equal in both groups (odds ratio 1.14; 95 % CI 0.36–3.56; *p* = 0.81). All predefined secondary endpoints including number of new lesions and total lesion volume on T2-weighted images, total number of new Gd-enhancing lesions on T1-weighted images, total brain volume, volume of grey matter, volume of white matter, EDSS, MSFC, relapse rate, time to first relapse, number of relapse-free patients and neutralizing antibodies did not show any significant differences (all *p* values >0.1). Transient elevations of liver enzymes were more frequent with atorvastatin (*p* = 0.02). In conclusion, atorvastatin 40 mg/day in addition to interferon beta-1b did not have a beneficial effect on relapsing-remitting MS compared to interferon beta-1b monotherapy over a 12-month period.

**Electronic supplementary material:**

The online version of this article (doi:10.1007/s00415-012-6513-7) contains supplementary material, which is available to authorized users.

## Introduction

Statins are lipid-lowering drugs inhibiting the 3-hydroxy-3-methylglutaryl-coenzyme A (HMG-CoA-) reductase, the main regulatory enzyme of cholesterol biosynthesis. In addition, statins have anti-inflammatory and immunomodulatory properties independent of their cholesterol-lowering effects [1].

Multiple sclerosis (MS) is a chronic inflammatory disorder of the central nervous system involving autoimmune mechanisms [2]. Some years ago, interest into statins for treatment of MS arose. Statins improve the course of experimental allergic encephalomyelitis (EAE), an animal model of MS [3–6]. Other experimental studies suggest a negative impact of statins on oligodendrocytes and myelin formation with impaired remyelination [7, 8]. Several clinical studies of different statins in different dosages given alone or in combination with interferon beta (IFNB) for relapsing-remitting MS (RRMS) yielded beneficial, harmful, or no effects as summarized in Table 1, whereas the largest trial of simvastatin as add-on therapy to interferon beta-1a (SIMCOMBIN) showed no beneficial effect [9–15]. However, to date, it is not clear whether statins have a class effect in MS or other statins in addition to disease modifying drugs might be beneficial or even harmful.

**Table 1:** Overview of clinical studies evaluating the combination of IFNB and statins in RRMS

	Study type	Patients	Allocation	IFNB	Statin	Primary endpoint/results
Paul et al. [10]	Open-label baseline-to-treatment trial	RRMS (*n* = 41)	IFNB + statin (*n* = 16) Statin (*n* = 25)	IFNB-1a 22 μg t.i.w. or IFNB-1b e.o.d.	Atorvastatin 80 mg/day	Trend towards reduction of Gd-enhancing lesions with IFNB + atorvastatin (*p* = 0.060)
Birnbaum et al. [12]	Double-blind, placebo-controlled trial	RRMS (*n* = 26)	IFNB + placebo (*n* = 9) IFNB + statin (*n* = 17)	IFNB-1a 44 μg t.i.w.	Atorvastatin 80 mg/day (*n* = 10) or 40 mg (*n* = 7)	Increased MRI and clinical disease activity with atorvastatin (*p* = 0.019)
Rudick et al. [13]	Post hoc analysis	RRMS (*n* = 582)	IFNB (*n* = 542) IFNB + statin (*n* = 40)	IFNB-1a 30 μg once weekly	Atorvastatin or simvastatin	No difference in annualized relapse rate and secondary endpoints
Lanzillo et al. [14]	Longitudinal controlled trial	RRMS (*n* = 45)	IFNB (*n* = 24) IFNB + statin (*n* = 21)	IFNB-1a 44 μg s.c. t.i.w.	Atorvastatin 20 mg/day	Fewer Gd-enhancing lesions versus baseline (*p* = 0.007) and fewer relapses versus the two pre-randomization years (*p* < 0.001) with atorvastatin
Togha et al. [11]	Double-blind, randomized controlled trial	RRMS (*n* = 80)	IFNB + placebo (*n* = 38) IFNB + simvastatin (*n* = 42)	IFNB-1a 30 μg once weekly	Simvastatin 40 mg/day	Lower relapse rate with simvastatin (*p* = 0.01)
Sörensen et al. [15]	Placebo-controlled randomized trial	RRMS (*n* = 307)	IFNB + statin (*n* = 151) IFNB + placebo (*n* = 156)	IFNB-1a 30 μg once weekly	Simvastatin 80 mg/day	No difference in annualized relapse rate and secondary endpoints
SWABIMS	Randomized controlled trial	RRMS (*n* = 76)	IFNB + statin (*n* = 38) IFNB (*n* = 38)	IFNB-1b e.o.d.	Atorvastatin 40 mg/day	No difference of patients with new T2-lesions and in secondary endpoints

*n* number, *IFNB* interferon beta, *t*.*i*.*w*. three times per week, *e*.*o*.*d*. every other day

In the SWiss Atorvastatin and Interferon Beta-1b trial in MS (SWABIMS) we evaluated the efficacy, safety, and tolerability of atorvastatin 40 mg per os (p.o.) daily and subcutaneous (s.c.) interferon beta-1b (IFNB-1b) every other day (e.o.d) compared to monotherapy with s.c. IFNB-1b e.o.d., an established therapy for RRMS [16].

## Materials and methods

### Study design

**Fig. 1:**
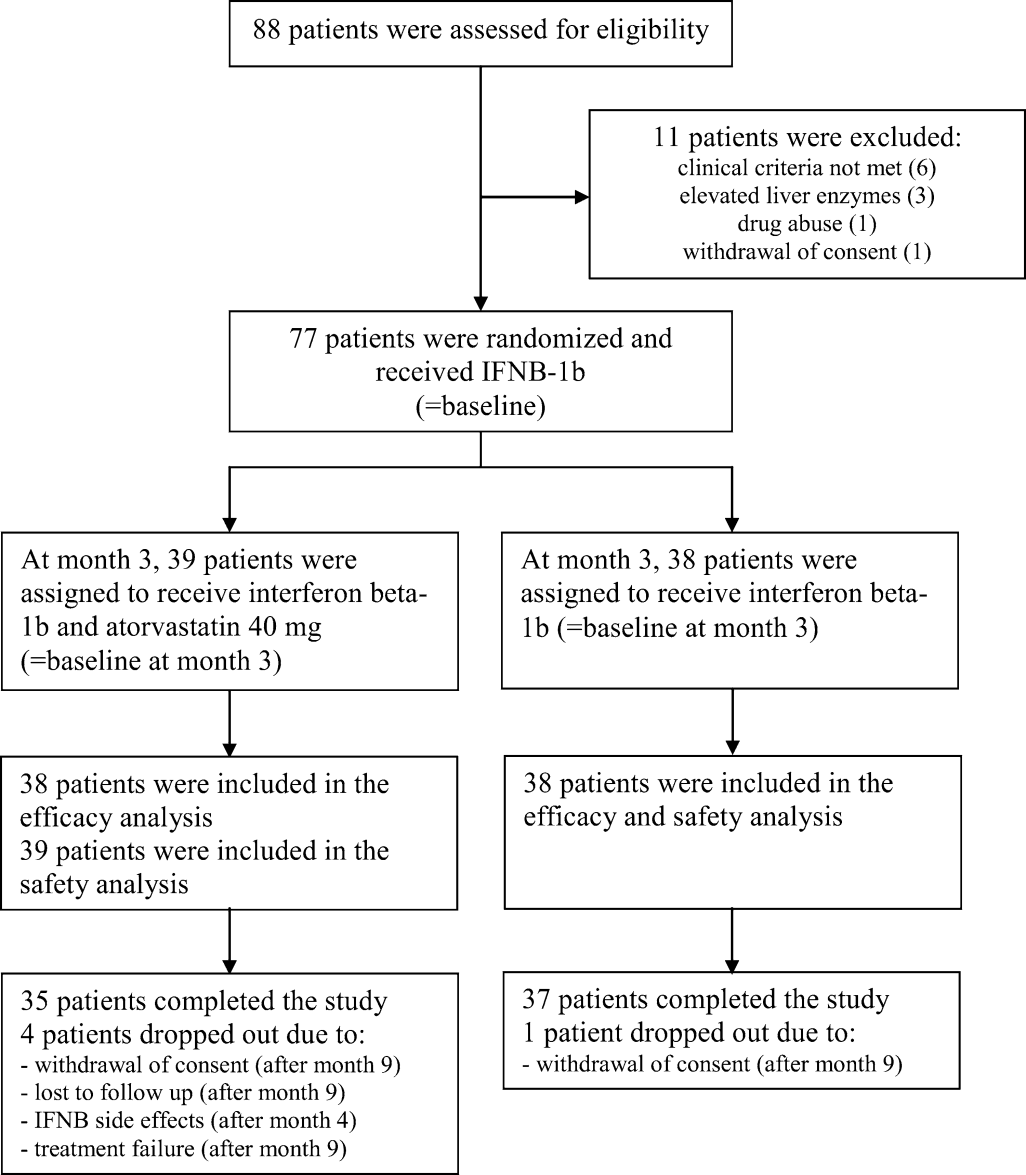
Enrollment, allocation, and follow-up of patients

SWABIMS was a multi-center, randomized, parallel-group, rater-blinded study in eight Swiss hospitals [17]. At the beginning of the study (termed “baseline”), all patients started IFNB-1b (Betaferon^®^/Betaseron^®^, Bayer Schering Pharma) for 3 months (termed “monotherapy phase”). At month three (termed “baseline at month three”), they were randomized 1:1 to receive atorvastatin 40 mg/day or not in addition to IFNB-1b for another 12 months (termed “randomized phase”) (Fig. 1).

For the primary endpoint and all clinical and radiological secondary endpoints, data at month 15 were compared to data at baseline at month three before randomization to atorvastatin or not.

Randomization was performed centrally by the clinical research organization (CRO) after baseline visit in four-block size, according to the randomization list (atorvastatin “yes” or “no”) generated with “RANCODE Professional 3.6” [18].

Patients and treating physicians were aware, whether atorvastatin was added. Placebo was not dispensed. Examining physicians scoring disability [Expanded Disability Status Scale (EDSS); Multiple Sclerosis Functional Composite (MSFC)] and neuroradiologists evaluating magnetic resonance images (MR) were blinded to treatment assignments [19, 20].

Atorvastatin was chosen because of its potent anti-inflammatory and immunomodulatory properties and favorable safety and pharmacokinetic profile [21–23]. Statins may cause dose-dependent elevation of hepatic enzymes [24]. Therefore, the use of atorvastatin 40 mg/day in combination with the potentially also hepatotoxic IFNB-1b was reasonable, especially since the optimal immunomodulatory dose of statins in MS is unknown.

Each patient had to provide written informed consent prior to study entry. The study was conducted in accordance with the International Conference on Harmonisation Guidelines for Good Clinical Practice (1996) and the Declaration of Helsinki (2006), and was approved by the local ethics committees and Swissmedic [25, 26]. The trial Registration Identifier is 2005DR2119 (Swissmedic) and NCT00942591 (clinicaltrials.gov).

### Patients

Patients with RRMS according to the 2005 McDonald’s criteria and disease duration >3 months, at least one relapse in the past 2 years, ≥ three lesions on spinal or brain-MR or both, baseline EDSS score from 0 to 3.5 (inclusive), and age from 18 to 55 years were enrolled.

Main exclusion criteria were primary or secondary progressive MS, clinically isolated syndrome (CIS), previous therapy with monoclonal antibodies, mitoxantrone, other cytotoxic or immunosuppressive drugs, and IFNB or glatiramer acetate within the last 12 months.

### Study endpoints

The primary endpoint was the proportion of patients with new lesions on T2-weighted MR images at month 15 compared to baseline at month three.

Secondary endpoints were the number of new lesions on T2-weighted images, change in total lesion volume on T2-weighted images (burden of disease), total number of new gadolinium (Gd-)-enhancing lesions on T1-weighted images, changes in total brain volume, volume of grey matter and volume of white matter, clinical disease progression (EDSS, MSFC), relapse rate, time to first relapse, number of relapse-free patients, and neutralizing antibodies (NAbs).

Adverse events (AE), laboratory data, vital signs and concomitant medication were analyzed as safety variables.

### Study procedures

IFNB-1b was started at a dose of 0.0625 mg e.o.d. and then increased weekly by 0.0625 to 0.25 mg e.o.d for the baseline phase of 3 months.

At month three, atorvastatin 40 mg/day was given to patients randomized to the atorvastatin/IFNB-1b group for 12 months. The other patients continued with IFNB-1b monotherapy for the whole study period.

Regular visits were performed at month one, three, four, six, nine, 12, and 15 for the assessment of EDSS, MSFC, NAbs, laboratory tests, MR, and efficacy and safety endpoints. Atorvastatin use was controlled by counting the returned tablets at visits at months six, nine, and 15. A patient was considered as compliant when he took at least 80 % of all atorvastatin tablets.

A relapse was defined as a newly appearing objective neurological abnormality in the absence of fever or known infection, lasting for at least 24 h and occurring at least 30 days after a preceding clinical event, correlating with the patient’s reported symptoms and increasing the total EDSS score or at least one of the functional systems of the EDSS score. Fatigue, mental, and/or vegetative symptoms were not classified as relapse.

Relapses were treated within 7 days with intravenous methylprednisolone 500 mg/day for 5 days followed by tapering-out with oral prednisolone.

MR scans were acquired on 1.5-Tesla scanners at screening, months three, nine, and 15. The MR protocol included T1-weighted axial spin-echo, T1-weighted sagittal 3D MPRAGE, axial dual-echo, i.e., proton-density, T2-weighted turbo-spin-echo images and axial T1-weighted spin-echo images after intravenous Gd injection.

MR scans were assessed centrally by two neuroradiologists at the Institute of Diagnostic and Interventional Neuroradiology of the University of Bern [27, 28]. A T2 lesion was defined as an area of increased signal on both the proton-density and the T2-weighted images. Disagreeing interpretations were discussed among the neuroradiologists to reach consensus. The image processing was performed with an algorithm enabling semi-automatic volumetry [29].

Laboratory analyses except NAbs were performed by Viollier AG. Atorvastatin was reduced to 20 mg/day in case of a more than threefold increase and stopped in case of more than fivefold increase of transaminases. Afterwards liver enzymes were controlled regularly and atorvastatin was continued when transaminases were below a threefold increase.

NAbs were assessed at the Ospedale San Luigi, Orbassano, Italy. The cytopathic effect assay was used as recommended by the World Health Organization [30]. Data from the neutralization assay were reported as reciprocal of the highest dilution of serum inducing 50 % neutralization. The neutralization titer was calculated according to Kawade’s formula and expressed in laboratory units (LU). A concentration of >20 LU/ml was considered positive [31]. Patients with one or more NAb-positive titers were defined NAb-positive. Two centers did not collect NAbs, explaining the lower numbers of individuals for this analysis.

### Statistical analysis

SAS version 9.2 was used for all statistical analyses. To obtain a power of 84 % to detect the difference between the monotherapy group proportion, π1, of 0.610 and the combination therapy group proportion, π2, of 0.910 with a 0.05 two-sided significance level in the Fisher’s exact test, a sample size of 38 patients in each group was needed [32]. All patients who took at least one dose of study medication and had at least one follow-up observation were analyzed [Full Analysis Set (FAS)]. Missing data because of drop-outs on the primary endpoint were replaced with MR data from the last available examination, which was month nine in all drop-outs. The same approach was used for other efficacy endpoints. Missing values for other parameters were treated as missing, except for severity and relationship of AEs to study drugs, which was regarded as severe and related to study drug.

Categorical data were described by frequency and percentage, continuous data by mean, standard deviation, minimum, 1st quartile, median, 3rd quartile, and maximum. Hypothesis tests were carried out with a α-level of 0.05, two-sided. All inferential analyses were presented by* p* values, point estimations and two-sided 95 % CI for treatment differences. If the assumption of normality in the linear models was not fulfilled, transformations of the data or non-parametric approaches like the Wilcoxon signed-rank test were used.

Differences between treatment groups at baseline were tested using *t* test or Fisher’s exact test depending on the distribution of the data.

The primary efficacy variable was the proportion of patients with new T2 lesions at month 15 compared to baseline at month three. Based on a logistic regression model with the factors treatment and gender and the covariates number of T2 lesions, number of Gd-enhancing lesions, EDSS, relapse rate and time since MS diagnosis at baseline at month three, the two-sided hypothesis of equality between the two treatments was tested at an α-level of 0.05. The results were presented as odds ratios and the associated two-sided 95 % CI and* p* values. Furthermore, a Fisher’s exact test for proportions was executed to test for the unadjusted treatment effect.

Secondary efficacy variables were analyzed with covariance, logistic regression models, or Fisher’s exact test depending on the distribution. Time to first relapse was analyzed with non-parametric methods for failure time data (Wilcoxon test) and illustrated by a Kaplan–Meier plot.

Assessments of safety and tolerability variables were presented by treatment group. AEs were summarized for each treatment group by presenting the number and percentage of subjects having an event, the number and percentage of event in each system organ class and preferred term, as well as severity and relationship to the study drug.

Any medication taken during the study was classified as concomitant and coded using WHO-Drug 2007.1.

## Results

The recruitment period was from May 2005 to December 2008, in which 88 patients were screened. Seventy-seven patients fulfilled the study criteria and were included and all of them were randomized at baseline at month three. None of them had previous immunomodulatory or immunosuppressive therapy. Five patients dropped out, four in the atorvastatin/IFNB-1b and one in the IFNB-1b group (Fig. 1). The EDSS score of one patient at screening was too high as we realized only in retrospect. This patient was excluded from the efficacy analysis (76 patients) but remained in the safety analysis (77 patients). The atorvastatin compliance was >80 % in the randomized phase. All relapses were treated with steroids as defined above.

Demographic and baseline characteristics are presented in Table 2. Patients of the atorvastatin/IFNB-1b group were younger than patients of the IFNB-1b group. Gender, ethnic origin, height, weight, and BMI were well matched.

**Table 2:** Patient characteristics

Characteristics	Atorvastatin/interferon beta-1b	Interferon beta-1b	*p* value
	*n* = 38	*n* = 38	
Demographic characteristics at screening/baseline			
Age (years)			
Mean ± SD	30.5 ± 7.9	35.7 ± 7.95	0.0032
Median (range)	28 (19 to 50)	36 (18 to 49)	
Gender (*n*, %)			
Male	17 (44.7 %)	15 (39.5 %)	
Female	21 (55.3 %)	23 (60.5 %)	0.82
Caucasian (*n*, %)	38 (100 %)	38 (100 %)	
Height (cm)			
Mean ± SD	171.8 ± 8.30	170.6 ± 9.75	
Median (range)	170 (156 to 191)	166.5 (157 to 192)	0.34
Weight (kg)			
Mean ± SD	71.42 ± 14.98	72.48 ± 18.18	
Median (range)	70 (43 to 106)	69 (44.5 to 124)	0.91
BMI (kg/m^2^)			
Mean ± SD	24.15 ± 4.61	24.74 ± 5.3	
Median (range)	23.39 (16.4 to 34.6)	23.07 (17.2 to 45.5)	0.70
MR findings at baseline at month 3			
No. of T2 hyperintense lesions			
*n*	36	37	
Mean ± SD	27.3 ± 24.24	21.2 ± 19.24	
Median (range)	21 (1 to 113)	14 (2 to 80)	0.19
Total volume of T2 hyperintense lesions (cm^3^)			
*n*	36	37	
Mean ± SD	3.5 ± 3.24	2.7 ± 2.68	
Median (range)	2.96 (0.3 to 12.4)	1.69 (0.1 to 9.6)	0.22
No. of GD-enhancing lesions on T1-weighted images			
*n*	37	38	
Mean ± SD	1.1 ± 2.9	0.3 ± 0.64	
Median (range)	0 (0 to 17)	0 (0 to 2)	0.08
Total volume of GD-enhancing lesions on T1-weighted images (cm^3^)			
*n*	37	38	
Mean ± SD	0.09 ± 0.21	0.02 ± 0.07	
Median (range)	0 (0 to 1.1)	0 (0 to 0.4)	0.043
Total brain volume (cm^3^)			
*n*	28	31	
Mean ± SD	1,476.8 ± 161.9	1,418.6 ± 151.97	
Median (range)	1,431.6 (1,209 to 1,898)	1,411.4 (1,129 to 1,782)	0.16
Volume of grey matter (cm^3^)			
*n*	28	31	
Mean ± SD	734.6 ± 68.09	708.7 ± 73.29	
Median (range)	728.97 (620 to 867)	694 (587 to 880)	0.14
Volume of white matter (cm^3^)			
*n*	28	31	
Mean ± SD	434.5 ± 46.29	416.3 ± 65.2	
Median (range)	428.5 (366 to 573)	427.1 (270 to 544)	0.21
Clinical characteristics			
MS duration at screening (years)			
Mean ± SD	0.88 ± 2.86	0.86 ± 1.46	0.20
No. of relapses in the past 2 years before screening (*n*, %)			
*n*	38	38	
1	12 (31.6 %)	11 (28.9 %)	
2	19 (50.0 %)	24 (63.2 %)	
3	3 (7.9 %)	3 (7.9 %)	
4	3 (7.9 %)	0 (0 %)	
8	1 (2.6 %)	0 (0 %)	0.38
EDSS at baseline at month 3			
*n*	38	38	
Mean ± SD	1.8 ± 1.0	1.9 ± 1.1	
Median (range)	2 (0 to 3.5)	2 (0 to 4)	0.37
MSFC at baseline at month 3			
*n*	38	38	
Mean ± SD	0.26 ± 0.49	0.18 ± 0.46	
Median (range)	0.38 (−1.2 to 1.0)	0.18 (−0.9 to 1.0)	0.26

*n* number of patients, *SD* standard deviation, *EDSS* Expanded Disability Status Scale: *MSFC* Multiple Sclerosis Functional Composite, *BMI* body mass index: *ns* no significant difference

Mean duration since diagnosis of MS, relapse rate within the past 2 years, number and volume of lesions on T2-weighted images, number and volume of Gd-enhancing lesions on T1-weighted images, brain volume and EDSS and MSFC scores at baseline did not differ significantly. During the monotherapy phase, both groups developed equally regarding all endpoints with no statistically significant differences. At baseline at month three, there was a trend towards a higher disease activity of the atorvastatin/IFNB-1b group caused by the distribution at baseline and the decline of the arithmetic average, median, and variability.

The results for the primary and secondary efficacy variables are given in Table 3. The proportion of patients with new lesions on T2-weighted images at month 15 compared to baseline at month three was not different according to the logistic regression model (*p* = 0.81). The adjusted odds ratio (OR) and the 95 % CI for the treatment difference of atorvastatin/IFNB-1b versus IFNB-1b were 1.14 and 0.36–3.56. To test the unadjusted treatment differences, an exploratory analysis with Fisher’s exact test was performed. Again, no significant difference was detected (*p* = 0.64).

**Table 3:** Efficacy endpoints (FAS, *n* = 76)

Endpoint	Atorvastatin/interferon-beta-1b	Interferon-beta-1b	*p* value
	*n* = 38	*n* = 38	
MR endpoints			
Proportion of patients with new lesions on T2-weighted images, baseline at month 3 to month 15 (*n* %)			
*n*	37	37	
Yes	18 (47.37)	15 (39.47)	
No	19 (50.0)	22 (57.89)	
Odds ratio for atorvastatin/IFNB-1b versus IFNB-1b (95 % CI)	1.14 (0.366 to 3.56)		0.81
No. of new lesions on T2-weighted images, baseline at month 3 to month 15			
*n*	36	37	
Mean ± SD	3.3 ± 6.81	1.7 ± 4.05	
Median (range)	0 (0 to 36)	0 (0 to 21)	
Treatment difference for atorvastatin /IFNB-1b vs. IFNB-1b (95 % CI)	−0.45 (−2.12 to 1.22)		0.59
Change in lesion volume (cm^3^) on T2-weighted images, baseline at month 3 to month 15			
*n*	36	37	
Mean ± SD	0.4 ± 2.65	0.2 ± 1.26	
Median (range)	0 (−4 to 12)	0 (−1 to 5)	
Treatment difference for atorvastatin /IFNB-1b vs. IFNB-1b (95 % CI)	−0.50 (−1.21 to 0.19)		0.15
Total number of Gd-enhancing T1 lesions at month 9 and 15			
*n*	37	38	
Mean ± SD	3.9 ± 12.89	2.2 ± 5.41	
Median	0 (0 to 65)	0 (0 to 25)	
Treatment difference for atorvastatin /IFNB-1b vs. IFNB-1b (95 % CI)	−1.76 (−4.78 to 0.96)		0.20
Change of total brain volume (cm³), baseline at month 3 to month 15			
*n*	27	31	
Mean ± SD	−13.7 ± 59.32	−4.9 ± 33.7	
Median (range)	−3.7 (−295 to 36)	−2.7 (−108 to 115)	0.91
Change of grey matter volume (cm^3^), baseline at month 3 to month 15			
*n*	27	31	
Mean ± SD	−4.0 ± 18.2	−5.8 ± 41.95	
Median (range)	−0.5 (−58 to 31)	−1.5 (−185 to 101)	0.21
Change of white matter volume (cm^3^), baseline at month 3 to month 15			
*n*	27	31	
Mean ± SD	0.9 ± 12.35	2.5 ± 39.24	
Median (range)	1.5 (−28 to 26)	−0.7 (−141 to 126)	0.78
Clinical endpoints			
Change in EDSS score, baseline at month 3 to month 15			
*n*	37	38	
Mean ± SD	0.03 ± 0.90	0.17 ± 0.5	
Median (range)	0 (−2 to 2)	0 (−2 to 2)	
Least squares means for effect treatment (95 % CI)	−0.11 (−0.54 to 0.32)		0.61
Change in MSFC score, baseline at month 3 to month 15			
*n*	37	38	
Mean ± SD	0.1 ± 0.27	0.1 ± 0.32	
Median (range)	0.1 (0–1)	0.1 (−1 to 1)	
Least squares means for effect treatment (95 % CI)	−0.08 (−0.22 to 0.06)		0.24
Relapse, baseline at month 3 to month 15			
*n*	38	38	
Relapse-free (*n*, %)			
No	18 (43.4 %)	13 (34.2 %)	
Yes	20 (52.6 %)	25 (65.8 %)	
Odds ratio of atorvastatin/IFNB-1b versus IFNB-1b (95 % CI)	0.65 (0.22 to 1.90)		0.43
No. of relapses			
Total number	28	23	
Mean ± SD	0.7 ± 0.98	0.6 ± 1.05	
Median (range)	0 (0 to 4)	0 (0 to 4)	0.63
Time to first relapse (25 % quartiles estimates)			
Mean ± SD	220.3 ± 18.08	284 ± 18.73	0.16
Neutralizing antibodies (NAb)			
NAb-positive (*n*, %)			
*n*	29	31	
No	13 (44.8 %)	20 (64.5 %)	
Yes	16 (55.2 %)	11 (35.5 %)	0.12
Change from NAb-positive to NAb-negative			
*n*	29	31	
No	14/16 (87.5 %)	6/11 (54.5 %)	
Yes	2/16 (12.5 %)	5/11 (45.5 %)	0.22

Treatment differences were calculated using ANCOVA

*n* number of patients with data, *SD* standard deviation, *EDSS* Expanded Disability Status Scale, *MSFC* Multiple Sclerosis Functional Composite

The predefined secondary endpoints number of new lesions and total lesion volume on T2-weighted images, total number of Gd-enhancing lesions on T1-weighted images, total brain volume, volume of grey matter, volume of white matter, EDSS, MSFC (including subscores), relapse rate, and number of relapse-free patients did not show any significant differences between the treatment groups at month 15 (all *p* values >0.1). In individual patients, data on study endpoints were missing because of a variety of reasons, e.g., movement artifacts during single MR sequences or incomplete data collection at visits. Two centers did not provide adequate MRI data for the analysis of total brain volume and grey and white matter volumes. This explains the lower numbers of individuals in some endpoints.

The logistic regression model regarding the primary endpoint with new T2 lesions as dependent variable and treatment, number of T2 lesions, number of Gd-enhancing T1 lesions, volume of Gd-enhancing T1 lesions, relapse rate, EDSS, time since MS diagnosis, age and gender at baseline as influencing variables showed that age (*p* = 0.04), number of Gd-enhancing T1 lesions (*p* = 0.02) and number of T2 lesions (*p* = 0.01) at baseline had a significant influence on the number of new T2 lesions whereas treatment did not (*p* = 0.72). Furthermore, age had a significant influence on the dependent variables of relapse rate, total brain volume, and volume of white matter whereas treatment did not.

NAb were evaluated in 60 of 77 patients (29 in the atorvastatin/IFNB-1b group; 31 in the IFNB-1b group). Sixteen patients turned NAb-positive in the atorvastatin/IFNB-1b group and 11 patients in the IFNB-1b group (*p* = 0.12). Neither the time of occurrence of NAb nor the titers differed between the groups. Five of 11 patients in the IFNB-1b group and two of 16 patients in the atorvastatin/IFNB-1b group turned from NAb-positive to NAb-negative during the study (*p* = 0.22).

The time to first relapse failed to prove significance in the Wilcoxon test as well (*p* = 0.16). The median (50 % quartile) time to first relapse could be calculated for the atorvastatin/IFNB-1b group, but because of an insufficient number of relapses not for the IFNB-1b group. The 25 % quartiles (atorvastatin/IFNB-1b group 100 days; IFNB-1b group 220 days) showed a non-significant shorter time to the next relapse in the atorvastatin/IFNB-1b group.

The Cox regression model with the time to first relapse as dependent variable and treatment, gender, number of T2 lesions, number of Gd-enhancing lesions, EDSS, relapse rate, time since diagnosis, age and volume of T1 lesions as influencing variables showed that age (*p* = 0.04) had a significant influence on the time to first relapse whereas treatment did not (*p* = 0.33).

Details on AEs by system organ class are given in Table 4. During the monotherapy and randomized phases, any AEs including serious and severe AEs occurred equally in both groups. During the randomized phase, AEs were more frequently related to the study drug in the atorvastatin/IFNB-1b group.

**Table 4:** Adverse events by system organ class MedDRA (FAS, *n* = 77)

Events *n* (%)	Atorvastatin/ Interferon-beta-1b	Interferon-beta-1b	*p* value
	(*n* = 39)	(*n* = 38)	
Total number of adverse events	101	89	
Adverse events (AE) by number of subjects			
Overall adverse event	36 (92.3 %)	27 (71.1 %)	ns
Monotherapy phase			
Any AE	25 (64.1 %)	21 (55.3 %)	ns
Any serious AE	0 (0 %)	0 (0 %)	ns
Any severe AE	0 (0 %)	2 (5.3 %)	ns
Any AE related to study drug	17 (43.6 %)	17 (44.7 %)	ns
Any AE leading to discontinuation of study drug	0 (0 %)	2 (5.3 %)	ns
Randomized phase			
Any AE	31 (79.5 %)	24 (63.2 %)	ns
Any serious AE	0 (0 %)	1 (2.6 %)	ns
Any severe AE	1 (2.6 %)	2 (5.3 %)	ns
Any AE related to study drug	22 (56.4 %)	12 (31.6 %)	0.02
Any AE leading to discontinuation of study drug	0 (0 %)	1 (2.6 %)	ns
Most frequently (>5 %) reported AE during the randomized phase by number of subjects			
Eye disorders			
Glaucoma	0 (0 %)	2 (5.3 %)	ns
Gastrointestinal disorders			
Diarrhea	2 (5.1 %)	2 (5.3 %)	ns
Nausea	3 (7.7 %)	1 (2.6 %)	ns
General disorders/administration site conditions			
Fatigue	2 (5.1 %)	4 (10.5 %)	ns
Influenza-like illness	4 (10.3 %)	5 (13.2 %)	ns
Pyrexia	0 (0 %)	2 (5.3 %)	ns
Infections and infestations			
Influenza	3 (7.7 %)	3 (7.9 %)	ns
Nasopharyngitis	5 (12.8 %)	2 (5.3 %)	ns
Injury, poisoning, and procedural complications			
Joint injury	2 (5.1 %)	0 (0 %)	ns
Abnormal laboratory values			
Elevated liver enzymes	9 (23.1 %)	2 (5.3 %)	0.02
Musculoskeletal and connective tissue disorders			
Muscle spasms	2 (5.1 %)	4 (10.5 %)	ns
Myalgia	3 (7.7 %)	0 (0 %)	ns
Nervous system disorders			
Headache	3 (7.7 %)	4 (10.5 %)	ns
Muscular weakness	0 (0 %)	2 (5.3 %)	ns
Paraesthesia	1 (2.6 %)	2 (5.3 %)	ns
Psychiatric disorders			
Depression	2 (5.1 %)	2 (5.3 %)	ns
Renal and urinary disorders			
Bladder disorder	0 (0 %)	2 (5.3 %)	ns
Respiratory, thoracic and mediastinal disorders			
Epistaxis	0 (0 %)	2 (5.3 %)	ns
Pharyngolaryngeal pain	2 (5.1 %)	1 (2.6 %)	ns
Skin and subcutaneous tissue disorders			
Acne	2 (5.1 %)	1 (2.6 %)	ns
Dry skin	1 (2.6 %)	2 (5.3 %)	ns
Eczema	2 (5.1 %)	0 (0 %)	ns

*AE* adverse event, *n* number, *ns* no significant difference

In the randomized phase, elevated liver enzymes occurred more often in the atorvastatin/IFNB-1b group (*p* = 0.02). All other AEs were equally distributed. Because of elevated liver enzymes, atorvastatin was transiently reduced in six patients (mean 3.1 month) and stopped for good in three patients 3.6 month on average before study termination. In the IFNB-1b group, IFNB was stopped temporarily in one patient.

In the atorvastatin/IFNB-1b group, AEs were classified as mild in 16 (41 %), moderate in 14 (35.9 %), and severe in one (2.6 %) subject. The severe AE was an influenza-like illness. There was one serious AE (SAE), a lumbar herniated disk. In the IFNB-1b group, AEs were classified as mild in ten (26.3 %), moderate in 12 (31.6 %), and severe in two (5.3 %) subjects. The severe AEs were dermal herpes zoster and lumbar disk prolapse. Blood lipid levels were similar at baseline at month three. Total and low-density lipoprotein cholesterol decreased significantly (*p* < 0.0001) in the atorvastatin/IFNB-1b group compared to the IFNB-1b group.

## Discussion

Atorvastatin 40 mg added to IFNB-1b did not have any beneficial effect on RRMS compared to IFNB-1b monotherapy over a period of 12 months. There were no significant differences in the primary or secondary endpoints between the two treatment groups.

Patients in the atorvastatin/IFNB-1b group were significantly younger, showed a trend towards higher disease activity at baseline, and had significantly larger volumes of Gd-enhancing lesions on T1-weighted images. A multiple regression analysis showed that this imbalance at baseline, and not the different treatment, was responsible for the trends towards a higher disease activity of the atorvastatin/IFNB-1b group at study end. Therefore, a negative effect of atorvastatin cannot be concluded.

The combination of atorvastatin and IFNB-1b was well tolerated and did not cause unexpected or severe side-effects. However, elevated liver enzymes without clinical symptoms occurred more often in the atorvastatin/IFNB-1b group and led to a temporary reduction or stop of atorvastatin in several patients. It cannot be distinguished whether atorvastatin alone or the combination accounted for the elevated liver enzymes. Liver enzymes normalized and atorvastatin could be continued at full dosage in most patients. However, in three patients, atorvastatin had to be stopped. Other AEs were similar in both groups.

SWABIMS also addressed the question, whether atorvastatin had an impact on NAbs against INFB-1b. There was a trend towards a higher prevalence and longer persistence of NAbs in the atorvastatin/IFNB-1b group that might indicate a negative effect of atorvastatin on NAb formation. However, for the moment, this does not have clinical implications.

The results of SWABIMS suggest that atorvastatin 40 mg added to IFNB-1b has no beneficial effect on RRMS. The results of SWABIMS are similar to the results of the SIMCOMBIN trial, the largest randomized trial that added simvastatin to IFNB-1a in RRMS as well as to a post hoc analysis of the SENTINEL trial. None of the two studies showed any beneficial effect of statins [13, 15]. Therefore, neither atorvastatin nor simvastatin are to be recommended as an add-on therapy to IFNB.

A minimal beneficial or harmful effect of other combinations of statins and IFNB cannot be definitely excluded yet. Other trials have supported positive or negative effects of statins, but this would have to be proved in larger studies (Table 1). A marked effect, however, seems unlikely because of the results of the largest trial (SIMCOMBIN), our SWABIMS study, and the comparable immunomodulatory properties of the different statins in experimental studies [6, 33].

The rationale to combine immunomodulatory drugs with different mechanisms of action is to obtain additive anti-inflammatory effects. This is the case for statins and IFNB-1b in vitro. Both inhibit the proliferation of stimulated peripheral blood mononuclear cells, reduce the expression of activation-induced adhesion molecules on T cells, modify the T helper 1/T helper 2 cytokine balance, reduce matrix metalloproteinases (MMP) -9, and downregulate chemokine receptors on both B and T cells [33]. However, combination therapies may lead to antagonistic effects as well. Besides anti-inflammatory effects, statins also show proinflammatory properties such as interferon-γ production, inhibit STAT1 phosphorylation, which is an important signaling pathway for IFNB, and antagonize the inhibitory effect of IFNB on the proteolytic activity on MMP-2 and MMP-9 [33–35]. The antagonistic mechanisms could potentially explain the negative results of studies combining IFNB and statins.

Multiple sclerosis patients with vascular risk factors and vascular disease have a more rapid disability progression than MS patients without [36, 37]. Therefore, vascular risk factors and diseases should be treated as rigorously as in non-MS patients. Provided that liver enzymes are monitored, SWABIMS suggests that atorvastatin 40 mg can be used for vascular prevention in MS patients who need a lipid-lowering therapy.

There are limitations of the SWABIMS study. It was a multi-center, randomized, parallel-group, rater-blinded trial, but not placebo-controlled. At the time of study planning and initiation, an identical placebo was not available. Therefore, we chose a prospective randomized rater-blinded end-point study design. Nevertheless, the evaluating clinicians and neuroradiologists assessing MR endpoints were blinded. Other limitations are the sample size and that we chose a surrogate marker instead of a clinical endpoint as primary endpoint. However, sample size calculations with the limited data of statins in MS available in 2005 indicated that the patient numbers of SWABIMS could give meaningful results with a primary MR endpoint. Another limitation might be the dose of atorvastatin. In vascular disease, higher doses of atorvastatin are more effective than lower doses. However, the optimal immunomodulatory dosage is unknown and it is not certain that higher doses yield higher efficacy. Therefore, and for safety reasons, we chose a daily dose of 40 mg of atorvastatin.

In conclusion, atorvastatin 40 mg/day in addition to IFNB-1b did not have any beneficial effect on RRMS compared to IFNB-1b monotherapy over a period of 12 months. Therefore, adding atorvastatin 40 mg/day to IFNB-1b seems to be no treatment option for patients with RRMS.

### Electronic supplementary material

Below is the link to the electronic supplementary material.

Supplementary material 1 (DOC 214 kb)

## Appendix

### SWABIMS study group

#### Neurology

Aarau: G Schwegler, M Leichtle, B Kieser; Basel: L Kappos, L Achtnichts, Y Naegelin; Bern: H P Mattle, C P Kamm, B Rieder, S Humpert, C Lienert, O Findling, S Jung, S Kipfer, A Mugglin, J Sellner, S Wolff, I Greeve, V Blatter, I Beiser; Biel: F Donati, M Alfaro, M Braunschweig; Münsterlingen: F Müller, L Schelosky, K-W Stock, M Nuding; Luzern: M Müller, P Stellmes, T Treumann, P Wicki; St. Gallen: B Tettenborn, N Putzki, S Müller, T Hundsberger, D Schött; Zürich: J Waskönig, C Gübelin, S Kollias, N Goebels.

#### MR core laboratory

University Institute of Diagnostic and Interventional Neuroradiology, Inselspital, Bern University Hospital, and University of Bern, Switzerland: M El-Koussy, Y Burren, J Slotboom, M Zbinden, C Kiefer, F v Bredow, R Wiest, G Schroth.

#### Clinical research organization

PharmaPart Gmbh (Bahnhofstrasse 20, Postfach 173, 8800 Thalwil, Switzerland) was contracted for data management, data analysis, randomization, visit tracking, drug distribution, case report forms, and coding of adverse events.

#### Safety monitoring board

B Weinshenker, M Matiello, Rochester MN, USA.
